# Uterine tube evisceration during drainage tube removal – A rare case report

**DOI:** 10.1016/j.ijscr.2024.109685

**Published:** 2024-04-23

**Authors:** Wessam Taifour, Hasan Youssef, Yahia Ranjous, Ali Deeb, Aimar Khir Abo Moughdeb

**Affiliations:** a Obstetrics and Gynecology Hospital, Damascus University, Damascus, Syria; b Faculty of Medicine, Damascus University, Damascus, Syria

**Keywords:** Drainage tube, Evisceration, Herniation, Uterine tube, Infundibulum, Ampulla

## Abstract

**Introduction and importance:**

Drainage tubes are commonly used to remove unwanted fluid after surgery. However, they are not indicated in all situations, and there is no evidence to support their common utilization.

**Case presentation:**

A 31-year-old woman at 38 weeks of gestation with a history of five cesarean sections presented with lower abdominal pain following a tonic-clonic epileptic seizure. Emergency surgery was performed due to fetal distress, and the uterus was found to be ruptured. After delivering the baby and closing the uterus, a drainage tube was inserted into the pouch of Douglas. Two days after surgery, the right ampulla and infundibulum were eviscerated from the drain site during the drainage tube removal. A second surgery was performed to reduce the herniated uterine tube.

**Clinical discussion:**

Drainage tubes are typically easily removed without complications. Some reported complications related to drainage tube removal include herniation, anchoring and suctioning of the uterine tube to the drainage tube, knotting with the colonic epiploica, and fracturing and retraction of the drainage tube due to adhesions. To the best of our knowledge, this is the first reported case of uterine tube evisceration during drainage tube removal.

**Conclusion:**

Evisceration after drainage tube removal is very rare. We believe that this is the first report of immediate evisceration after the removal process. Such complications can be avoided with more restricted instructions for the use of drainage tubes and more researches on the reasons for these complications.

## Highlights

• Uterine tube evisceration after drainage tube removal is very rare.
• This is the first report of immediate evisceration after the removal process.
• Tube evisceration can be avoided with more restricted instructions for the use of drainage tubes.
• More researches are needed to detect the reasons for these complications.

## 1 Introduction

Drainage tubes are catheters inserted near surgical wounds to remove blood, pus, gas and other fluids that might accumulate in free spaces [1]. The iliac fossae are the most common sites for placement of drainage tubes [2].The accumulation sites depend on gravity and patient position. Therefore, the Douglas pouch is the most common site for fluid accumulation in the vertical position, whereas both subphrenic and Douglas pouches are common sites for accumulation in the horizontal position. The drainage tube can be removed when the daily fluids are less than 30 ml/day for two straight days, which is typically within three days after the surgery [1]. Drains are usually removed easily without complications, with only a few cases – perhaps due to medico-legal issues – reporting rare complications, such as herniation, evisceration, knotting to the drain tube and others [3,4].

In our case, we report the evisceration of the right ampulla and infundibulum out of the drain incision immediately after removal of the drainage tube. Our work has been reported in line with the SCARE criteria [10].

## 2 Case presentation

A 31-year-old woman, gravida 5, para 5 (G5P5), at 38 weeks of gestation, presented to the emergency department of hospital. The patient's chief complaint was lower abdominal pain following a tonic-clonic epileptic seizure. Her obstetric history revealed five previous cesarean sections, with the last one being three years ago. The patient was diagnosed with epilepsy seven years ago and treated with sodium valproate. The medication was discontinued in the seventh month of pregnancy, but the patient reported taking one pill a few hours before to the seizure episode.

Upon inspection, the patient was pale and distressed, with a slim and underweight appearance. Her blood pressure was 100/70 mmHg, and her pulse rate was 88 beats per minute. Abdominal palpation revealed tenderness with a decrease in the expected uterine fundal height. The pelvic examination was normal. Echography imaging showed normal amniotic fluid and placenta location, with a femur length of 64 mm (33 week), biparietal diameter of 85 mm (34 week), and fetal heart rate of 100 beats per minute. Considering the recent epileptic seizure, increased uterine contractions, patient's history of five cesarean sections, and fetal distress, the doctors decided to perform an emergency cesarean section.

**Fig. 1:**
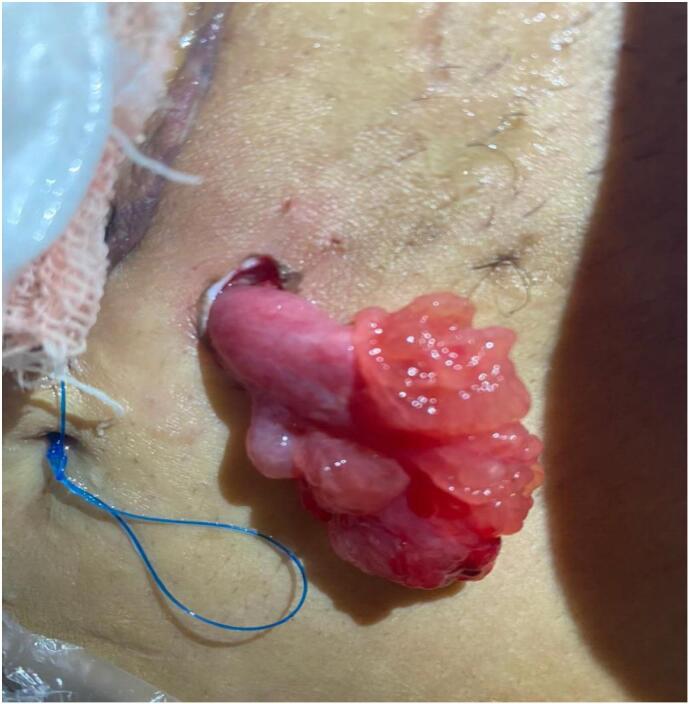
Ampulla and infundibulum herniated through the drainage incision.

The patient underwent an upper vertical incision, which revealed a ruptured uterus. During surgery, two units of blood and two units of plasma were transfused. A neonate weighing 1800 g was delivered, with a 1-min Apgar score of 4 and 5-min Apgar score of 6. The ruptured uterus was closed in two layers, and open drainage system with a 25 mm multichannel PVC drainage sheet was inserted in the right inguinal region. Two days after surgery, an unfortunate complication occurred during the removal of the drainage tube. The ampulla and infundibulum were stuck in the drainage tube, and during the removal process, they were pulled and herniated through a drainage incision (Fig. 1). Consequently, the patient was transferred for emergency surgery, and the previous incision was reopened. The surgical team attempted to reduce the herniated structures without breaching the fascia of Scarpa, but was unsuccessful. Therefore, the remaining layers were reopened and the hernia reduced, fallopian tube was not removed because patient refused tube removal. As a precautionary measure and because of adhesions, another drainage tube was inserted on the opposite side to remove excess fluid and blood to increase healing and decrease the chance of infection. The patient was closely monitored in the hospital for a few days before discharge.

## 3 Discussion

Drainage tubes are commonly used after standard surgeries, including abdominal surgeries [1]. While most drains are safe, they may still lead to various complications, such as vessel or tissue injury, infections, difficulties in removal, hernias and eviscerations [5]. It has been reported that 0.65 %–2.5 % of patients may experience viscera herniation from the port site after laparoscopy [6], with the small intestine being the most common organ to herniate [2]. Other organs that may herniate are the appendix, omentum, gall bladder, uterine tubes and ovaries [6].

Uterine tube herniation is a rare complication of drainage tube removal. Prior to this study, we found three cases of uterine tube herniation 24 h, 3 days and 4 days after drainage tube removal [2,6,7]. Two other cases reported anchored and suctioned uterine tubes that were detected before removal of the drainage tube [4,8]. Other reported complications include knotting with the colonic epiploica after emergency laparoscopy for an ectopic pregnancy [9], and fracturing and retraction of the drainage tube caused by adhesions [3]. To the best of our knowledge, this case is the first to report evisceration of the ampulla and infundibulum of the right uterine tube from the drain incision immediately after removal of drainage tube.

Risk factors for herniation include general weakness, inadequate nutritional status, obesity, increased intra-abdominal pressure chronic diseases (e.g., diabetes mellitus), steroid intake and stab incision [7].

The routine use of drainage tubes after surgery should be discouraged, and reserved for patient with expected complications [8]. When necessary, the diameter of the drainage tube should be less than 10 mm with few or even no side holes [2,8]. Some recommend that the distal tip should not be coiled around the pouch of Douglas [8]. The insertion and removal techniques of drainage tubes are also important to avoid evisceration. During insertion, abdominal drainage should never be placed in the surgical incision because of the weakness of the surgical wound, and it is preferred to be passed obliquely so that the muscles can close the drainage tract [8]. During removal, negative pressure in the closed drainage system should be removed, and 360° rotations should be applied to the drainage tube before withdrawing it. Forceful pulling out should be avoided when there is resistance, with the considering surgical reopening when attempts fail [8,9]. The material type of the drainage tube is still unknown whether it matters or not [8]. However, many studies that report herniation and evisceration have used different materials and systems [2,6,8].

In our patient, an open drainage system with a 25 mm multichannel PVC drainage sheet was used for prophylaxis. The patient was very slim and had general debility, with a history of five cesarean surgeries which led to weakness of the rectus abdominis muscle. In addition, a drainage tube was inserted vertically through the muscle in the pouch of Douglas. We believe that these are the potential reasons that contributed to the herniation. During removal, there was no negative pressure and the drainage tube was pulled out softly with no resistance. Fortunately, the ampulla and infundibulum herniated spontaneously and were immediately noticed. Therefore, the surgical team successfully managed to preserve the uterine tube.

## 4 Conclusion

Evisceration after drainage tube removal is very rare. We believe that this is the first report of immediate evisceration after the removal process. Such complications can be avoided with more restricted instructions for the use of drainage tubes and more researches on the reasons for these complications.

## Consent for publication

We got written informed consent from the patient to publish this article.

## Authors' information

Not applicable.

## Ethical approval

Not applicable.

## Funding

The authors did not receive funding for this study.

## Author contribution

HY and YR wrote the manuscript.

WT, AD and AA revised the manuscript.

All authors have read and approved the final manuscript.

## Guarantor

Wessam taifour.

## Research registration number

N/A.

## Conflict of interest statement

The authors declare that they have no competing interests.

## Data Availability

The laboratory tests results are available from the corresponding author on reasonable request.
